# Supplementary material for: Genome-wide screen of cell-cycle regulators in normal and tumor cells identifies a differential response to nucleosome depletion
Source: Cell Cycle. 2016 Dec 8;16(2):189–99. doi: 10.1080/15384101.2016.1261765 (PMC5283814; doi:10.1080/15384101.2016.1261765)
Supplement: Supplementary files [file kccy-16-02-1261765-s001.zip › 2016CC7212R-s02.pdf]

Supplementary Fig. S1. Sokolova et al.,2016

A

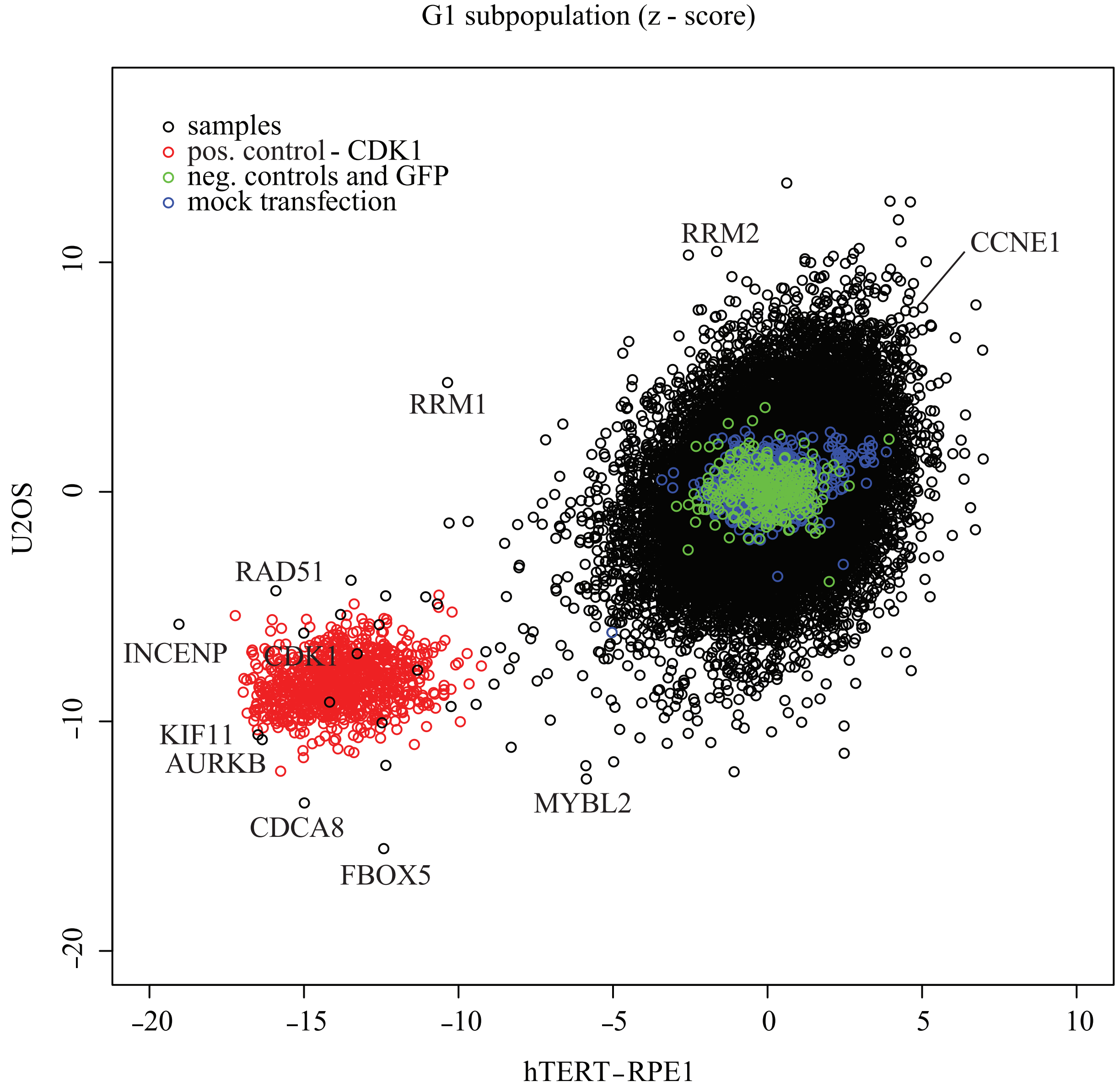

B

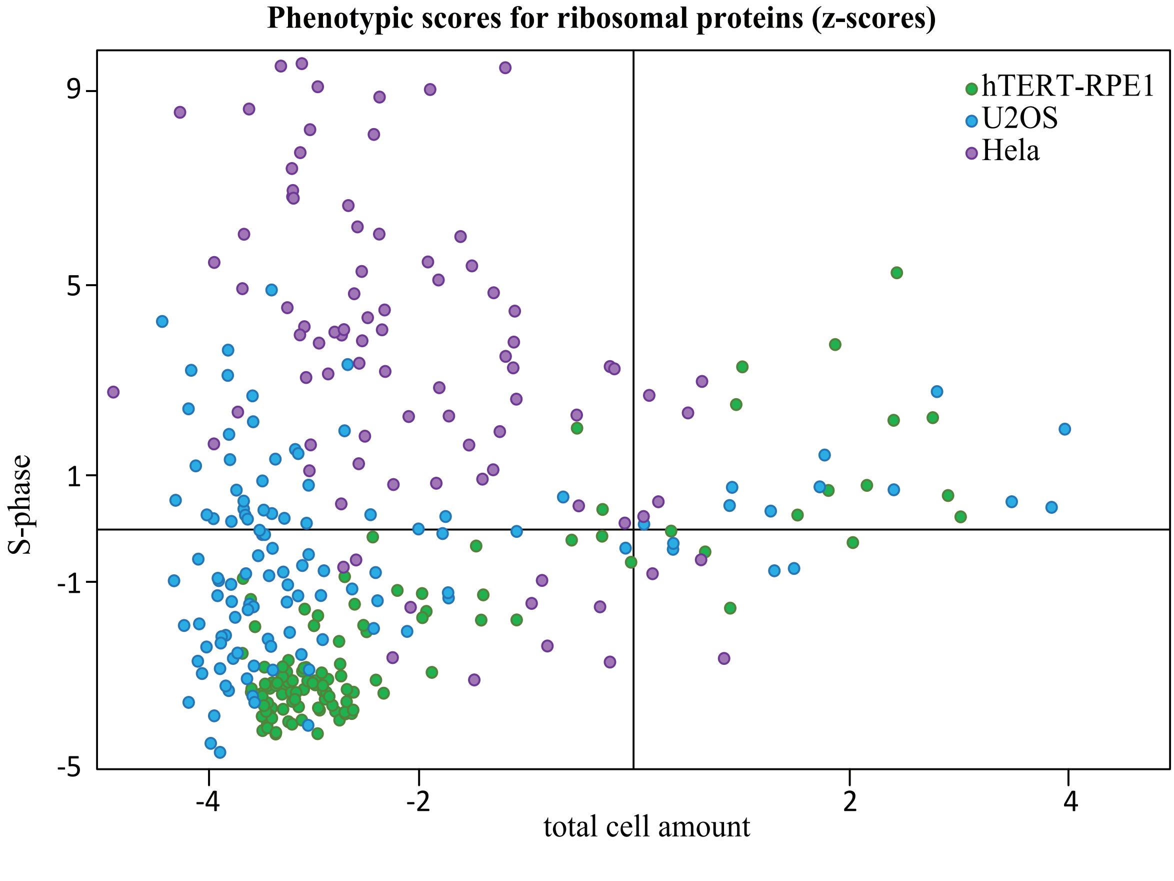

SUPPLEMENTARY FIGURE LEGENDS

Supplementary Figure S1: Genome-wide RNAi screen and secondary RNAi screen in several cancer cell lines. A, Genome-wide RNAi screen identified many common regulators that decrease G1 content in human immortalized (hTERT-RPE1) and cancer (U2OS) cell lines. Samples (black circles) and three groups of controls (colored circles) are shown. Known cell cycle regulators are indicated in black typeface. **B**, Z-scores of S-phase phenotype and total cell numbers from hTERT-RPE1 (green), U2OS (blue) and Hela (purple) cell lines for ribosomal protein genes. In hTERT-RPE1 (wt p53) knockdown of most ribosomal protein genes results in reduced amount of total cells and cells in S-phase, in Hela where p53 in not active - in increase amount of cell in S-phase (Hela data are from Kittler et al[14])

Supplementary Fig. S2. Sokolova et al.,2016

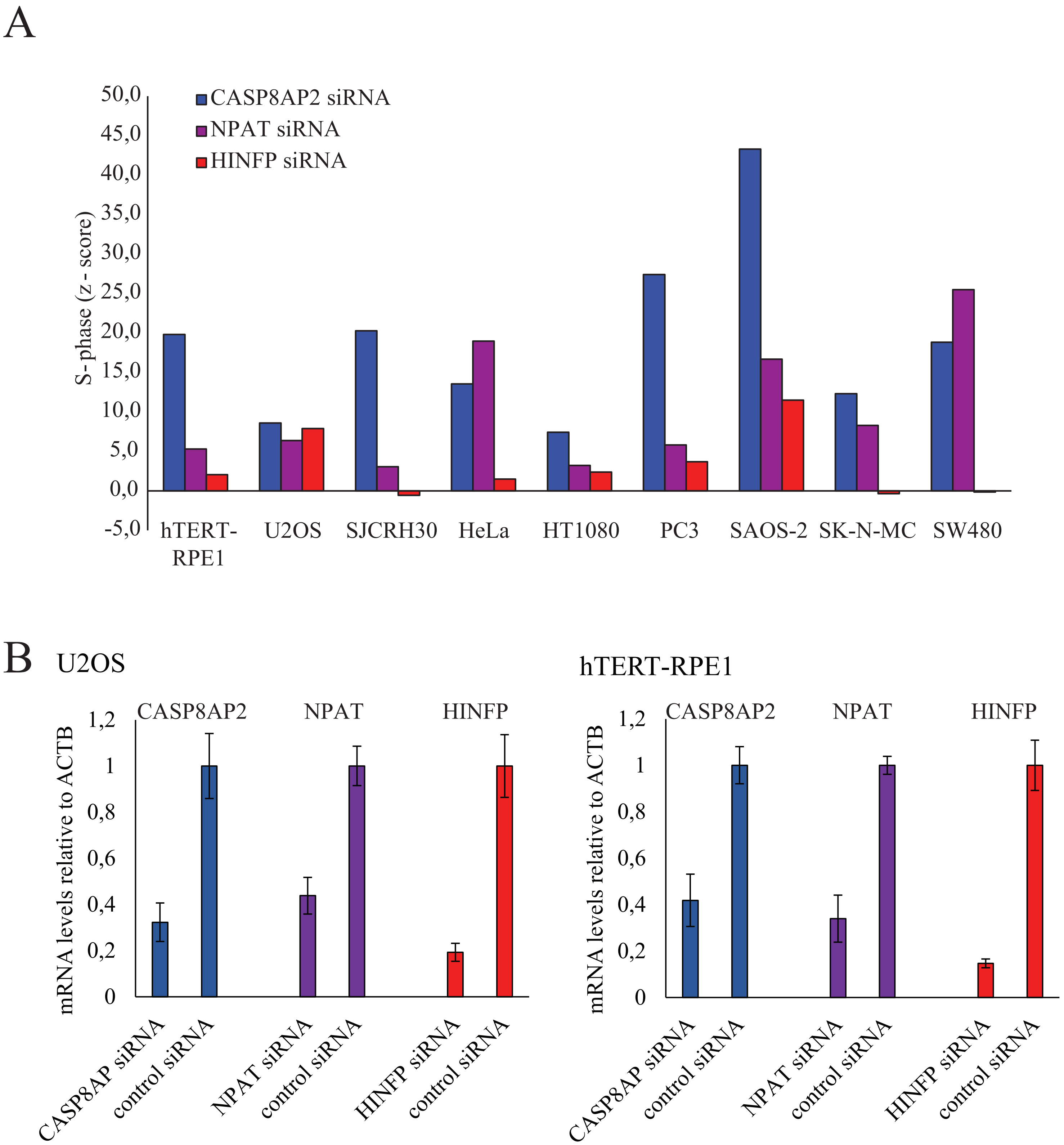

Supplementary Figure S2: CASP8AP2, NPAT and HINFP depletion in different cell lines. A, Secondary RNAi screen in different cancer cell lines shows significant S-phase arrest following CASP8AP2 knockdown in all cell lines; NPAT and HINFP knockdown have similar albeit weaker effect in most of cell lines. **B**, mRNA levels were measured by qPCR 3 days after transfection of U2OS and hTERT-RPE1 cells. Beta-actin was used as internal control, and the standard deviation was calculated from three independent experiments

Supplementary Fig. S3. Sokolova et al.,2016

A hTERT-RPE1

B U2OS

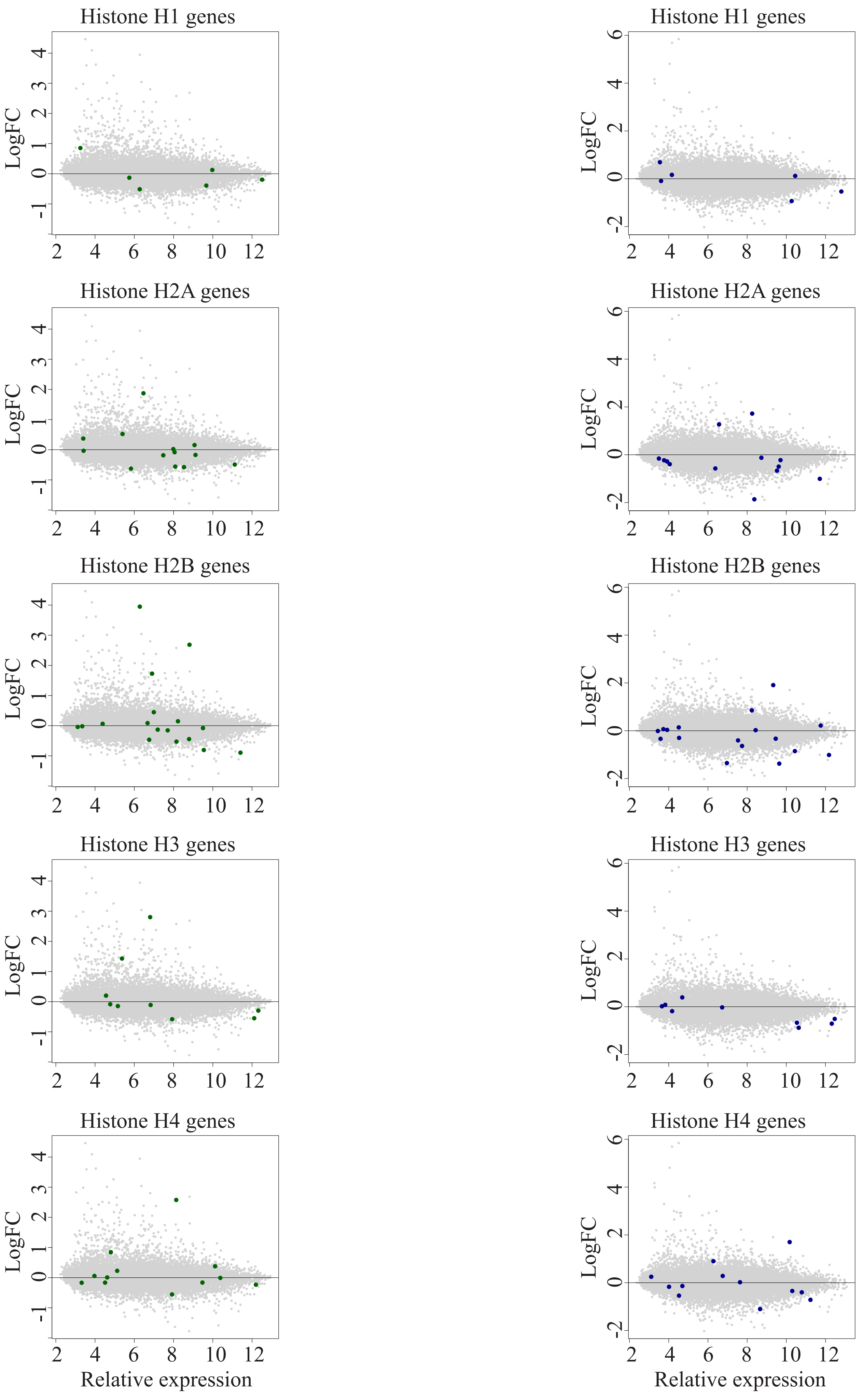

Supplementary Figure S3: Expression of different replication-dependent histone genes and changes following CASP8AP2 depletion in U2OS and hTERT-RPE1 cells. A, B, Microarray data of relative expression level (x-axis) and logFC (y-axis) 3 days after depletion of CASP8AP2 in hTERT-RPE1 (A) and U2OS (B) cells. Green dots indicate histone protein genes in hTERT-RPE1 cells and blue – in U2OS cells.

Supplementary Fig. S4. Sokolova et al.,2016

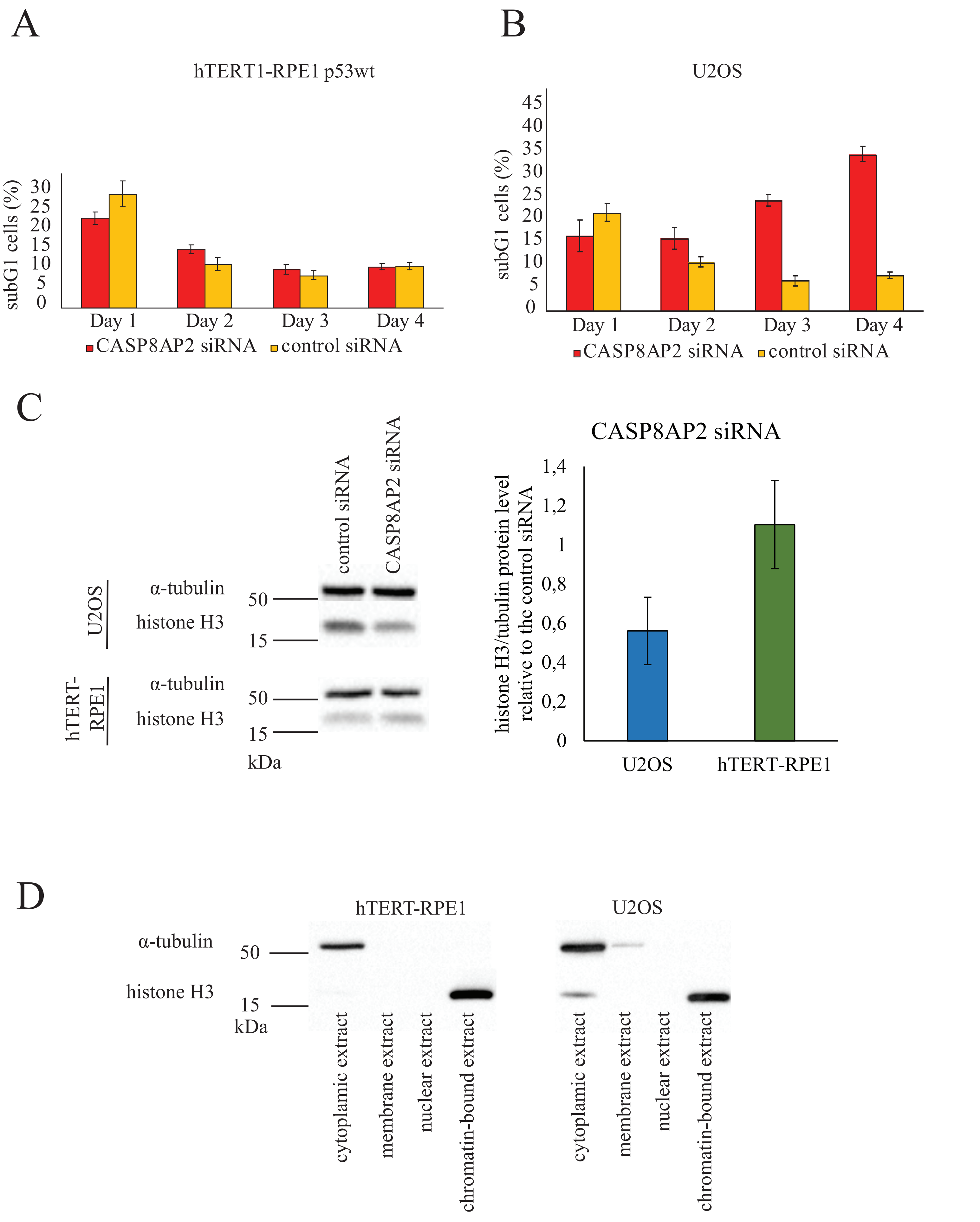

Supplementary Figure S4: Cell death is increased and protein levels of histone H3 are decreased in U2OS cells following CASP8AP2 depletion. A, B, Flow cytometry analysis of sub-G1 population measuring cell death in siRNA treated hTERT-REP1 (A) and U2OS (B) cells. **C**, Western-blot analysis of H3 following subcellular protein purification indicates that most of the H3 in both cells lines is chromatin-bound. **D**, Western blot analysis confirms decreased level of histone H3 after CASP8AP2 knockdown in U2OS but not in hTERT-RPE1 cell line. Mean of three independent experiments are shown. Error bars represent one standard deviation.

Supplementary Fig. S5. Sokolova et al.,2016

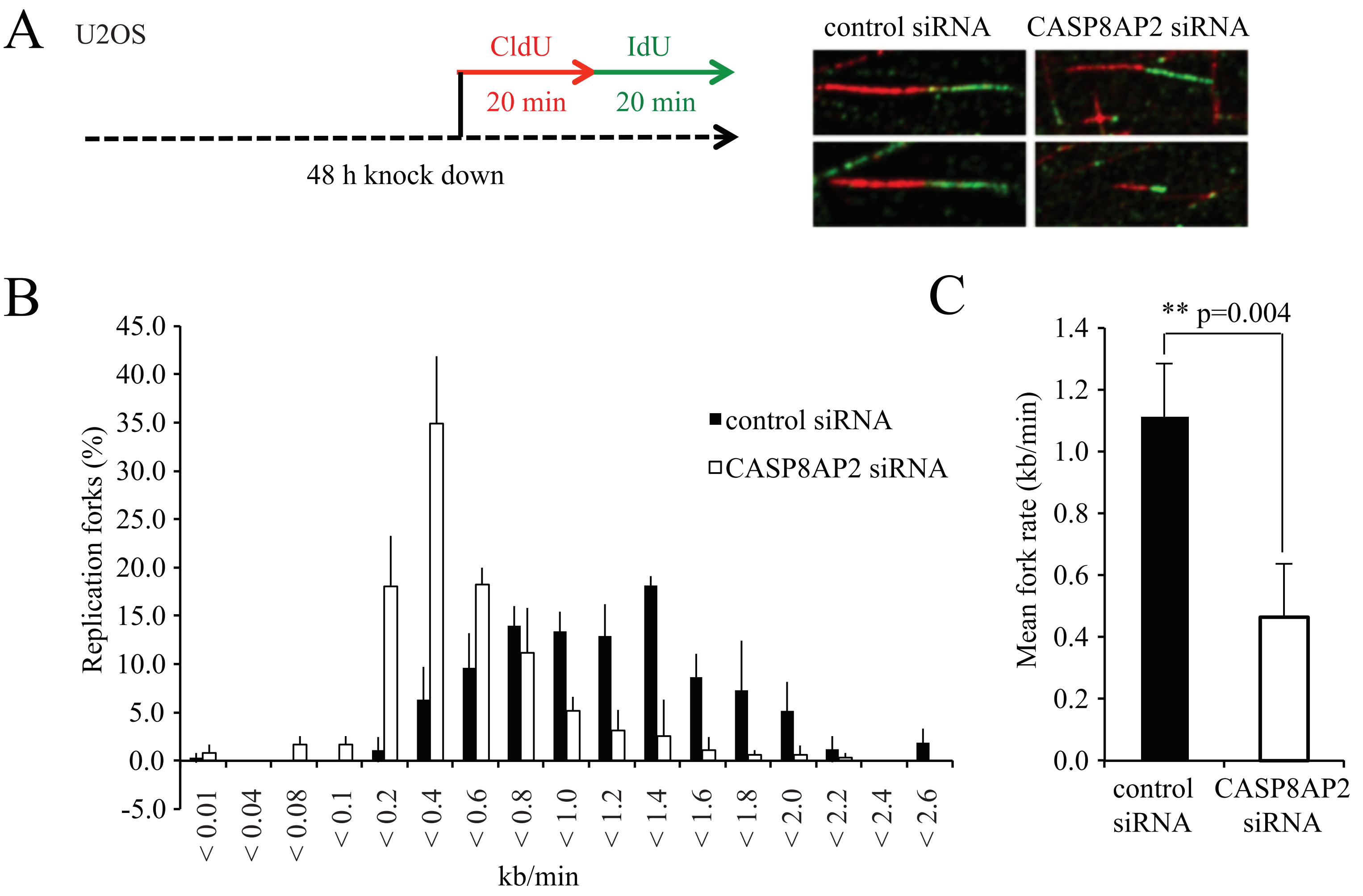

Supplementary Figure S5: Slower replication fork progression in U2OS cell line following CASP8AP2 depletion. **A**, Schematic representation of the DNA fiber assay measuring the length of labeled DNA strands. Images show representative DNA fibers 2d after control or CASP8AP2 siRNA transfection. **B**, Distribution of replication fork speeds in control and CASP8AP2 depleted cells. **C**, Significant reduction of mean fork rate in CASP8AP2 siRNA treated cells. Mean of three independent experiments are shown. Error bars represent one standard deviation.

Supplementary Fig. S6. Sokolova et al.,2016

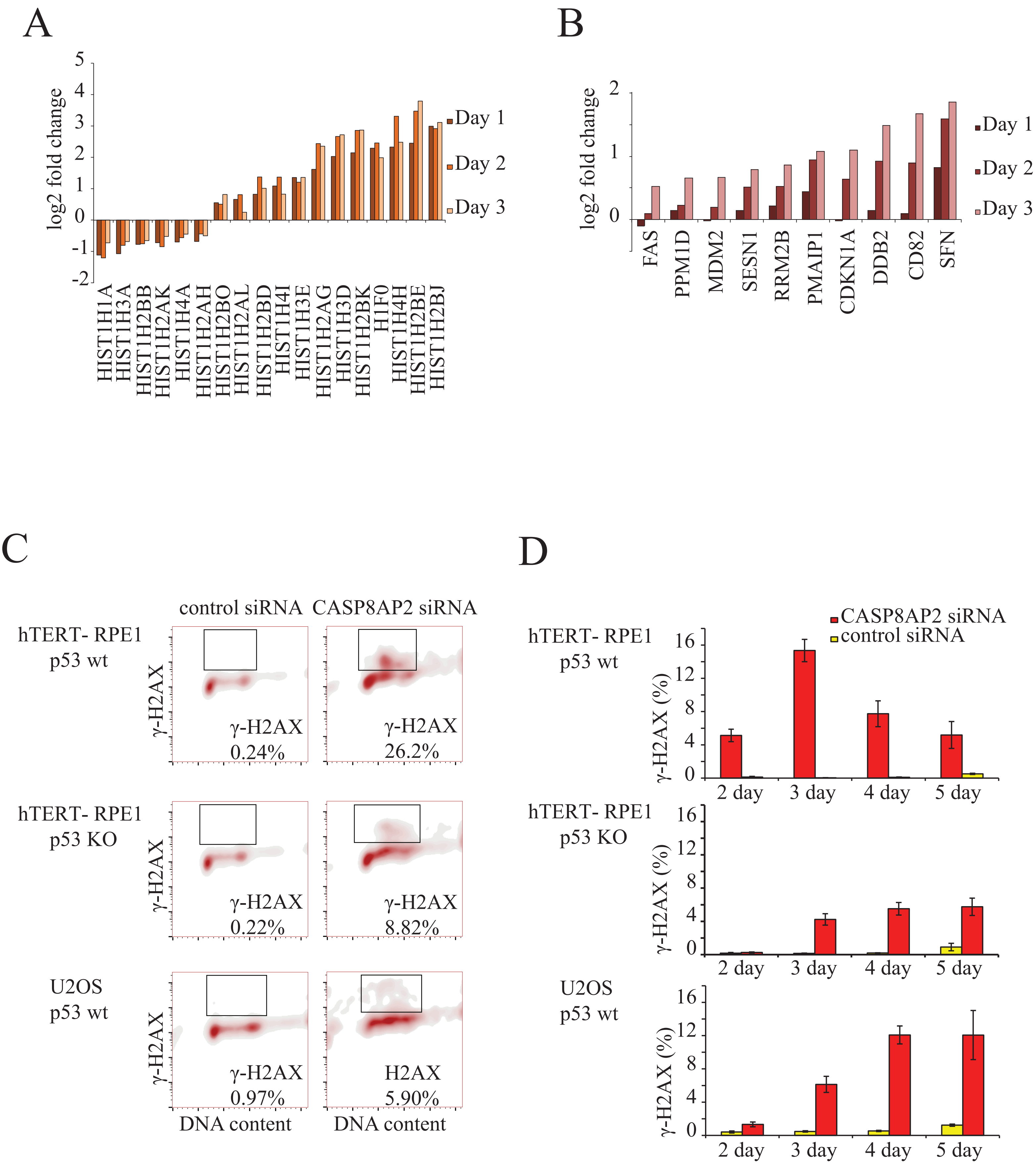

Supplementary Figure S6: H2AX Ser139 phosphorylation and p53 independent regulation of histone gene expression and DNA replication following CASP8AP2 knockdown. A, B, Histone gene deregulation precedes activation of p53. Microarray data in hTERT-RPE1 cells during three days following CASP8AP2 knockdown shows deregulation of (A) histone gene expression from the first day and (B) most of p53 target genes only after second day. **C**, H2AX Ser139 phosphorylation in S-phase arrested cells following CASP8AP2 knockdown in hTERT-RPE1 cell lines with different p53 status and U2OS cells 3 d after transfection. Note that DNA damage signaling is strongest in hTERT-RPE1 p53 wt cells. **D**, Different accumulation of γ-H2AX in CASP8AP2 depleted cancer and normal cells with different p53 status. Mean and standard deviation for biological triplicates are shown. Note that DNA damage signaling is highest in normal p53 wt cells at 3 days, decreasing at later time points. In contrast, in tumor cells and normal cells lacking p53 the response is initially weaker, but continues to increase at least up to 5 days.

Supplementary Fig. S7. Sokolova et al.,2016

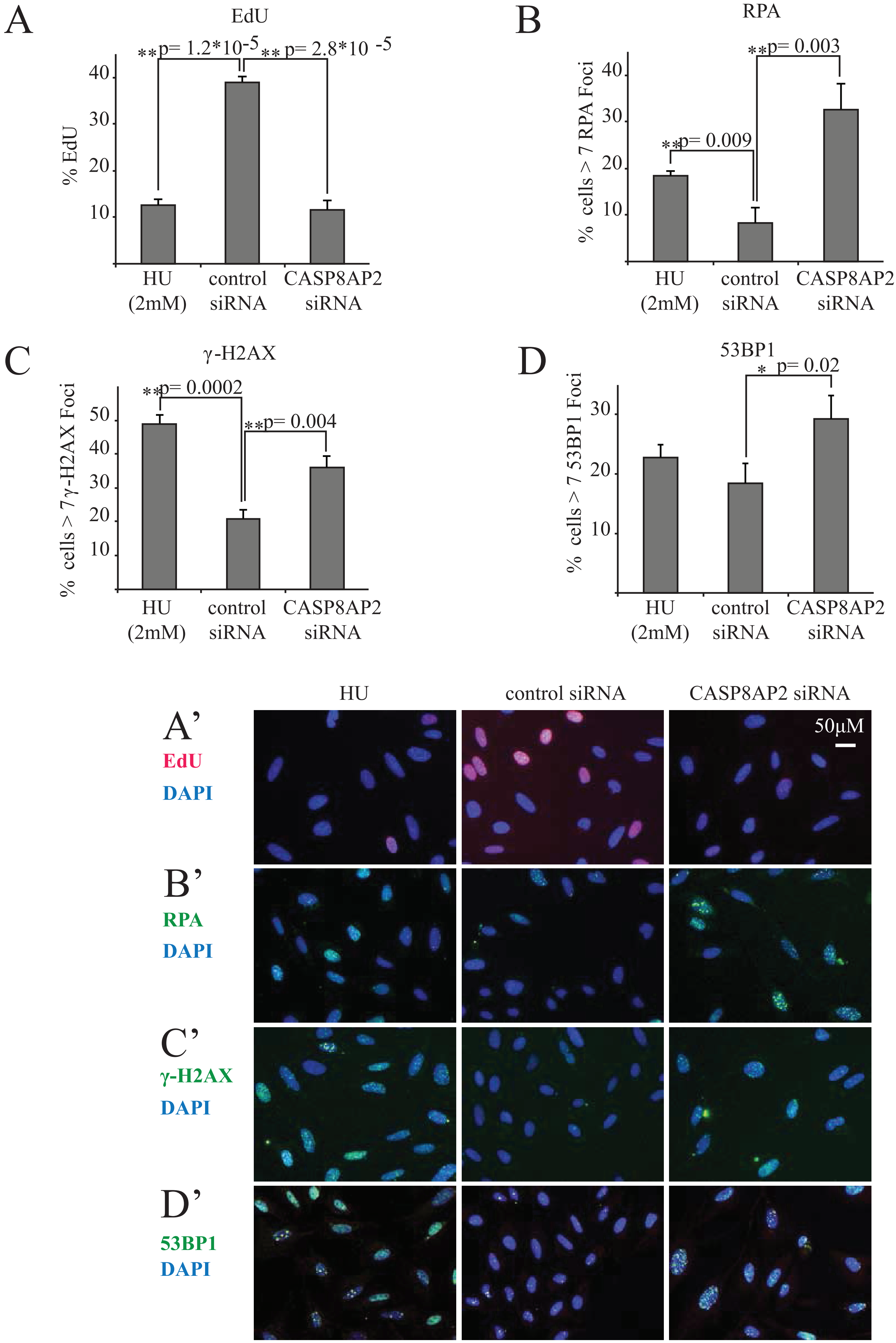

Supplementary Figure S7: CASP8AP2 is required to maintain genome integrity in U2OS. (**A**, **A'**) The defect in S-phase progression in CASP8AP2 deficient cells is comparably strong to hydroxyurea treatment as determined by EdU incorporation. (**B**, **B'**) Loss of CASP8AP2 results in accumulation of ssDNA (>7 RPA foci per cell) and increased DNA damage signaling measured by the recruitment of γ-H2AX (**C**, **C'**) and p53BP1 (**D**, **D'**). For all experiments (n=3) means and s.e.m are plotted and representative images are shown. P-values (*) are calculated with Student's t-test and scale bars represent 50 µm.

Supplementary Fig. S8. Sokolova et al.,2016

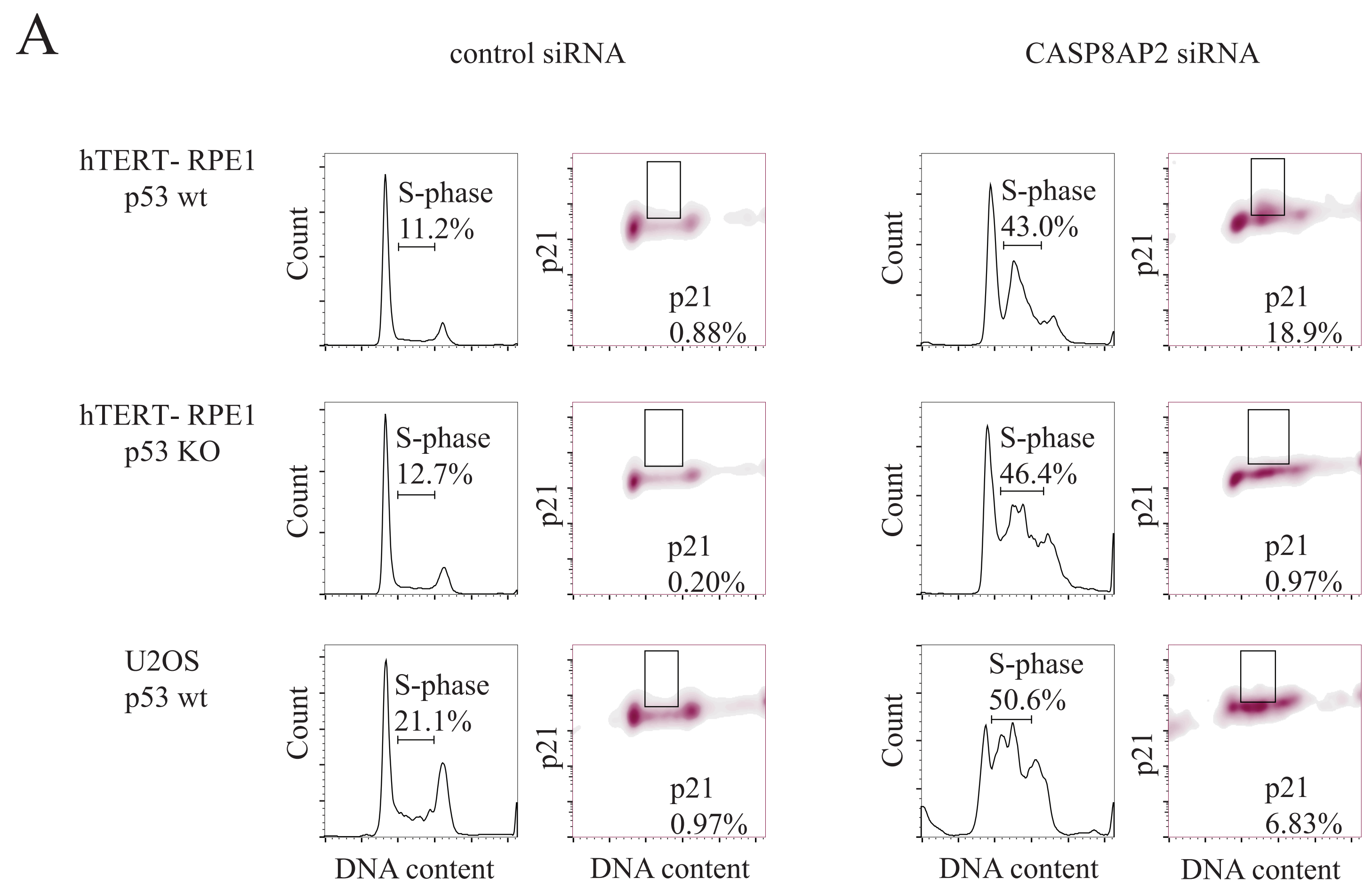

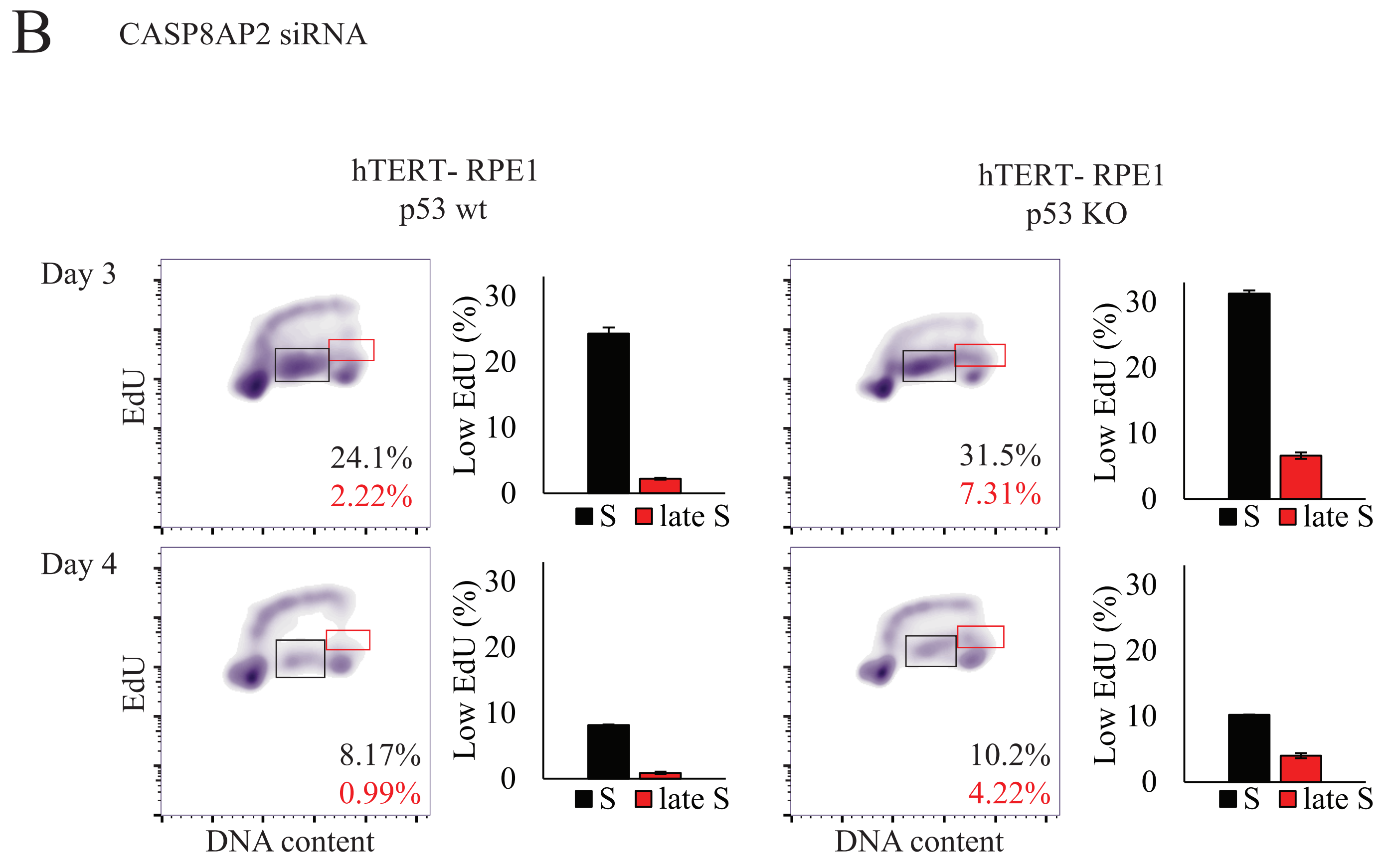

Supplementary Figure S8: p21 activation and DNA replication progression in CASP8AP2 depleted cells. A, Normal hTERT-RPE1 cells with wild-type p53 respond to CASP8AP2 knockdown by upregulating p21. Panels show flow cytometry analysis of p21 protein expression following CASP8AP2 knockdown in hTERT-RPE1 cell lines with different p53 status and U2OS cells. **B**, hTERT-RPE1 cells lacking p53 continue to replicate their DNA despite knockdown of CASP8AP2. EdU cell proliferation assay indicates that more DNA synthesis occurs in late S-phase hTERT-RPE1 p53 KO than hTERT-RPE1 p53 wt cells. Percentage of the cells with low EdU incorporation in mid-S and late-S phases is indicated black and red, respectively. Mean of three independent experiments are shown. Error bars represent one standard deviation.

Supplementary Fig. S9. Sokolova et

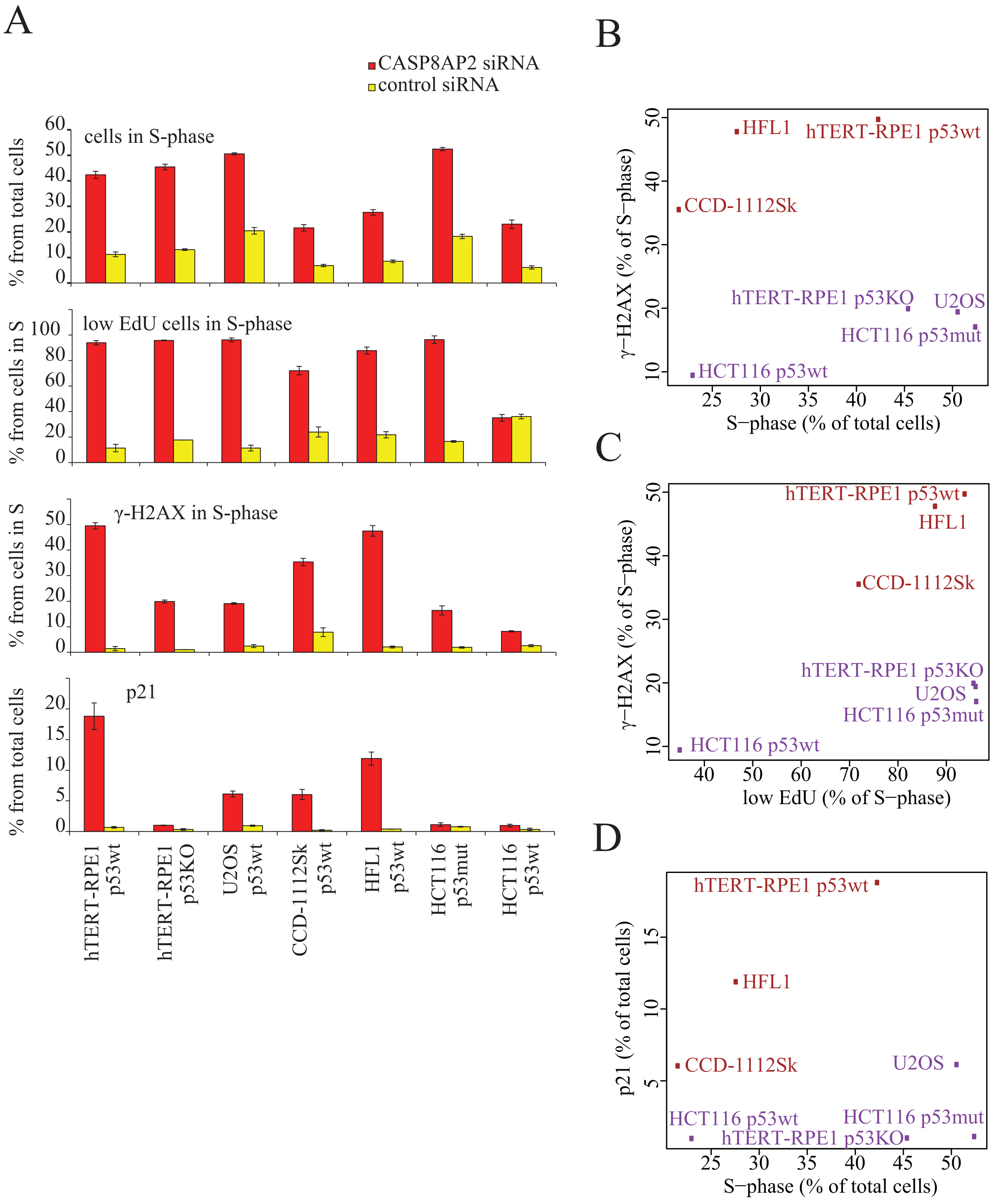

Supplementary Figure S9: Correlation of DNA replication progression, p21 accumulation and H2AX Ser139 phosphorylation in CASP8AP2 depleted cells. A, Number of cells in S-phase, EdU negative cells in S-phase, γ-H2AX cells in S-phase and p21 positive cells following CASP8AP2 knockdown in seven different cell lines. Mean and standard deviation for biological triplicates are shown. **B**, **C**, **D**, The correlation of amount of cells, EdU negative cells, γ-H2AX and p21 in S-phase for seven different cell lines (normal cells with p53wt are shown in brown). **B**, Accumulation of γ-H2AX in S-phase is higher for normal cell lines with p53wt than in cancer cells or normal cells with p53KO. **C**, Accumulation of γ-H2AX is higher in non-replicating or slowly replicating cells for normal cell lines with p53wt as compared to cancer cells or normal cells lacking p53. **D**, Only normal cells with wild-type p53 strongly upregulate p21 expression in S-phase in response to CASP8AP2 knockdown.
